# Olfactory impairment associated with reduced physical capacity 24 months after COVID-19

**DOI:** 10.1016/j.bbih.2025.101032

**Published:** 2025-06-13

**Authors:** Christoffer Granvik, Alicia Lind, Guilherme W.F. Barros, Clas Ahlm, Sara Anderson, Linus Andersson, Johan Normark

**Affiliations:** a Department of Clinical Microbiology, Umeå University, Umeå, Sweden; b Department of Diagnostics and Intervention, Umeå University, Umeå, Sweden; c Integrated Science Lab, Department of Physics, Umeå University, Umeå, Sweden; d Department of Epidemiology and Global Health, Umeå University, Umeå, Sweden; e Department of Psychology, Umeå University, Umeå, Sweden

## Abstract

**Background:**

Olfactory impairment has been associated with adverse health outcomes, particularly in older populations, including cognitive decline, malnutrition, and frailty. The COVID-19 pandemic highlighted olfactory impairment as a key symptom affecting individuals across all age groups, raising concerns about its long-term impacts. This study investigates the association between post-acute olfactory impairment and long-term physical capacity in COVID-19 patients, hypothesizing that impaired olfaction is linked to reduced physical performance.

**Methods:**

This prospective cohort study included 63 hospitalized and non-hospitalized COVID-19 patients (38.1 % women; median age 51 years, IQR 47.0–60.0) who underwent olfactory testing 1–3 months post-infection. Olfactory assessments included threshold screening, supra-threshold intensity ratings, and an odour identification test. Physical capacity was assessed using the 1-min sit-to-stand test at follow-ups (3, 6, 12, and 24 months). Partial correlation analysis and linear mixed models were used to analyse the data, adjusting for covariates such as age, sex, BMI, comorbidities, smoking status, and severity of infection.

**Results:**

In the early post-acute phase, 36.5 % of participants exhibited olfactory impairment. We identified a significant, negative correlation between objectively tested olfactory impairment and physical capacity at all follow-ups. In a linear mixed model adjusted for relevant covariates, olfactory impairment was associated with reduced physical capacity up to 24 months after infection. The association strengthened over time, reflected by the increasing beta values for the interaction term: 0.09 (p = 0.200) at 6 months, 0.13 (p = 0.053) at 12 months, and 0.23 (p = 0.001) at 24 months.

**Conclusion:**

Individuals with olfactory impairment in the early post-acute phase of COVID-19 infection were more likely to exhibit diminished physical capacity 24 months later. This study highlights the broader implications of olfactory impairment, previously noted mainly in older populations, demonstrating its relevance across age groups. The COVID-19 pandemic presented a unique opportunity to investigate this relationship, enhancing our understanding of how olfactory impairments relate to long-term physical performance. These findings emphasize the need for further research with larger, more diverse cohorts and objective longitudinal assessments to confirm and extend these observations.

## 1 Introduction

Reoccurring reports link olfactory impairment to different states of health impairments. A reduced sense of smell is a stronger predictor of mortality in elderly individuals than cardiovascular disease (Van et al., 2020). It is associated with cognitive dysfunction (Jacobson et al., 2024), dementia (Laukka et al., 2023), malnutrition (Olofsson et al., 2021), and physical frailty (Cheng et al., 2024; Yeo et al., 2024). Hyposmia (i.e., a reduced sense of smell) and anosmia (a complete lack of smell) both predicted impaired mobility in older adults, in terms of slower, and more rapid decline in walking speed across a 10-year period (Yuan et al., 2024). Further, when compared with normosmic (i.e., non-impaired olfaction) older adults, those with olfactory impairment were less likely to be able to complete a 400 m fast walking test (Yuan et al., 2024).

Following the COVID-19 pandemic, the patterns relating olfaction and distress mainly seen among the elderly, became manifest also in younger age categories. Olfactory impairment constituted a hallmark symptom, especially for the alpha and delta variants of the SARS-CoV-2 virus (von et al., 2023), with estimated prevalence rate of 48 % (Saniasiaya et al., 2021). Like previous reports among the elderly, olfactory impairment was found to be associated with a host of negative outcomes. Individuals with COVID-19-related olfactory impairment performed worse on cognitive tasks (Vilarello et al., 2023), developed post-COVID to a greater degree (Granvik et al., 2024), and reported a lower quality of life (Winter et al., 2023).

In parallel, Pandey et al. (2024) highlighted the up surge in neurological diseases following historical pandemics and argued that impaired olfaction could be a marker for various forms of motor dysfunction such as Parkinson's disorder. However, they also noted the lack of studies on olfactory impairment and physical function in COVID-19 patients.

We therefore aimed to investigate the potential association between post-acute olfactory impairment and long-term physical capacity in a sample of both hospitalized and non-hospitalized individuals after up to two years after COVID-19 infection. While exploratory, we hypothesized that olfactory impaired individuals would perform worse on 1-min sit-to-stand tests across time, compared with the non-impaired.

## 2 Methods

### 2.1 Study design and patient cohort

The CoVUm Study is a prospective multicentre study, previously described in published articles (Granvik et al., 2024; Ahmad et al., 2023-). Study participants were prospectively enrolled between April 2020 and June 2021 from study sites in Örebro, Umeå, Västerås and Karlstad. All individuals were confirmed SARS-CoV-2 positive with a PCR-test. Exclusion criteria were inability to provide informed consent or inability to read or communicate in Swedish. Ethical approval was granted from the Swedish Ethical Review Authority, Uppsala, (approval number: 2020-01557). In total, the CoVUm study consisted of 579 individuals. We selected a sub-cohort of participants (N = 69) using convenience sampling at one site, Umeå University Hospital, to undergo olfactory testing between September and December 2020.

Participant data, including baseline characteristics, were entered in electronic case report forms, using REDCap electronic data capture tools, hosted by Umeå University (Harris et al., 2009/04). Data was exported from the database on June 26th, 2023.

Physical capacity was assessed at the follow-ups using the 1-min sit-to-stand test (Bohannon and Crouch, 2019). Oxygen saturation and pulse were measured with pulse oximeter before and after the test. The study participants were additionally asked to rate dyspnoea and leg muscle fatigue before and after physical activity on a scale 0 to 10, were 10 represented maximum fatigue or dyspnoea.

### 2.2 Olfactory assessment

We assessed olfactory threshold, odour identification, and self-rated olfactory acuity 1–3 months after infection to investigate different features of olfaction. Self-rated olfactory acuity was then monitored during follow-ups as a convenient way to track improvement in the cohort.

#### 2.2.1 Olfactory threshold screening, and supra-threshold intensity ratings

Participants underwent an olfactory assessment including a threshold screening test, a supra-threshold assessment of intensity, and an odour identification test conducted in the early post-acute phase (one to three months after infection). The olfactory assessment is previously described (Granvik et al., 2024). In short, the threshold screening test was a two-alternative forced-choice test comprising blank and ascending concentrations of olfactory stimuli (*n*-butanol diluted in water to 0.04 mg/m3 and 3.5mg/m3, corresponding to dilution steps 6 and 2 described in Cain et al. (1988)). Participants were regarded as normosmic if they could correctly separate five consecutive flasks at dilution step 6 from blank, hyposmic if they could separate five consecutive flasks at dilution step 2 from blank, and anosmic if they failed both these tasks. To further quantify the olfactory acuity of the participants, participants were asked to rate the odour intensity of all flasks (blank, dilution step 6 and 2) using a Borg CR-100 rating scale (Borg and Borg, 2002).

#### 2.2.2 Odour identification

Further, all participants completed the Scandinavian odour identification test (SOIT) (Larsson et al., 2004). The test includes 13 different odours, e.g. vanilla, orange, and bitter almond, all assumed to be well known by the participants. They were tasked to identify these odours, selecting a response from four alternatives each time. The number of correct identifications comprised a total test score, maximum score equals 13.

#### 2.2.3 Self-rated olfactory acuity

The participants attended follow-ups at 3 months, 6 months, 12 months and 24 months after initial infection. In order to follow subjective odour deficit, the participants were asked to assess their sense of smell and taste after infection at each follow-up. The assessment included four response options: (1) normal, (2) total loss of smell or taste, (3) fluctuating ability to perceive odours or tastes, and (4) partial recovery but not fully restored.

### 2.3 Statistical analysis

Data analysis was performed using R (4.2.0). Wilcoxon rank sum test was used to compare continuous variables between groups and Pearson's Chi-squared test or Fisher's exact test for categorical variables.

We first evaluated the Spearman correlation between measures of olfactory tests and outcomes from physical capacity, controlling for age, sex, BMI, Charlson comorbidity index and severity of initial infection. We selected olfactory intensity rating (dilution step 6) as a continuous variable for further analysis. P-values were corrected using Benjamini-Hochberg.

An initial linear mixed model was estimated that considered number of sit-to-stands as the response variable, the participants study ID as a random effect on the intercept and the following fixed effects: Timepoints (3, 6, 12, 24 months), Olfactory intensity rating (dilution step 6), Age, Sex, BMI, Severity of initial infection, Charlson comorbidities index, and Smoking status. Backwards selection was performed to remove non-significant fixed effects with a threshold p-value of 0.05, which resulted in the removal of Sex, Charlson comorbidities index, and Smoking status as covariates. At last, we considered a model that accounts for a possible interaction between timepoints and Olfactory intensity rating.

We used the R package “glmmTMB” (Brooks et al., 2017) for estimating the linear mixed models. Residuals from the models were assessed with the R package “DHARMa” (Hartig, 2022).

## 3 Results

### 3.1 Study cohort

In the sub-cohort that underwent olfactory testing, six individuals were excluded, resulting in 63 individuals included in the study. Reasons for exclusion included one participant who dropped out before the 3-month follow-up, two who became pregnant during the follow-up period, and three who did not complete any 1-min sit-to-stand tests. The number of participants that completed physical assessment at each follow-up was; 57 at 3 months, 56 at 6 months, 58 at 12 months and 47 at 24 months. The cohort consisted of 24 women (38.1 %), and the median age was 51 years (47.0–60.0). No significant differences in baseline characteristics (Supplementary Table 1).

### 3.2 High prevalence of objective and subjective olfactory impairment after COVID-19

In the early post-acute phase after infection 40 (63.5 %) individuals were normosmics, 20 (31.7 %) were hyposmics, and three (4.8 %) anosmics according to the olfactory threshold screening test. During follow ups, study participants reported an improved sense of smell. This was reported in decreasing numbers, 30 individuals (49.2 %) experienced reduced sense of smell at 3 months, 21 (33.9 %) at 6 months, 20 (32.3 %) at 12 months and 14 (25.5 %) at 24 months. Study design and unadjusted values for number of sit-to-stands are summarized in Supplementary Fig. 1.

### 3.3 Persistent olfactory impairment correlates with reduced physical capacity

The olfactory threshold screening and intensity rating measures at early post-acute phase were significantly correlated to number of sit-to-stands at all follow-ups, Table 1. A notably strong correlation was, for instance, revealed for the rated intensity of *n*-butanol at dilution step 6 and number of sit-to-stands at 3, 6, 12, and 24 months, with correlation coefficients ranging from 0.45 to 0.56 (all p < 0.001). The odour identification test was not significantly correlated to any outcomes. Self-reported olfactory acuity was correlated with physical capacity only at the six-month follow-up. Change in pulse at 6 months and 24 months was significantly correlated with odour threshold screening tests at post-acute phase and, at six months, to subjective odour deficit. Change in saturation, reported dyspnea and leg fatigue during the physical capacity test were not significantly correlated to any measures after correction for multiple testing.

**Table 1:** Spearman's correlation analysis of olfactory tests and outcomes from physical capacity assessment at follow-up up to 24 months after COVID-19. Spearman's rho (p-value). Controlling for age, BMI, sex, hospitalization and Charlson comorbidity index. Significant p-values (<0.05) are written in bold, corrected using Benjamini-Hochberg.

**Outcomes**		**Odour detection threshold test**	**Scandinavian odour identification test**	**Intensity rating, blank**	**Intensity rating, dilution step 6**	**Intensity rating, dilution step 2**	**Subjective odour deficit at follow-ups**
	**Number of sit-to-stands at 3 months**	**−0.36 (0.038)**	0.19 (0.336)	**0.39 (0.020)**	**0.45 (<0.001)**	**0.49 (<0.001)**	−0.27 (0.176)
	**Number of sit-to-stands at 6 months**	**−0.56 (<0.001)**	0.23 (0.242)	0.3 (0.114)	**0.56 (<0.001)**	**0.57 (<0.001)**	**−0.44 (0.008)**
	**Number of sit-to-stands at 12 months**	**−0.54 (<0.001)**	0.18 (0.357)	0.25 (0.222)	**0.54 (<0.001)**	**0.65 (<0.001)**	−0.32 (0.078)
	**Number of sit-to-stands at 24 months**	**−0.46 (0.008)**	0.26 (0.242)	0.28 (0.222)	**0.56 (<0.001)**	**0.58 (<0.001)**	−0.23 (0.267)
	**Delta pulse at 3 months**	−0.08 (0.752)	0.09 (0.721)	0.17 (0.371)	0.08 (0.763)	0.11 (0.621)	−0.14 (0.500)
	**Delta pulse at 6 months**	**−0.46 (<0.001)**	0.2 (0.301)	0.04 (0.877)	**0.38 (0.020)**	**0.38 (0.020)**	**−0.42 (0.008)**
	**Delta pulse at 12 months**	−0.21 (0.267)	0.25 (0.235)	−0.08 (0.752)	0.23 (0.242)	0.2 (0.291)	−0.18 (0.336)
	**Delta pulse at 24 months**	**−0.5 (<0.001)**	0.3 (0.176)	−0.1 (0.714)	0.35 (0.080)	**0.48 (0.008)**	−0.24 (0.261)

We further analysed the association between olfactory impairment and physical capacity in a linear mixed model where the odour intensity rating showed a significant association with number of sit-to-stands at follow-ups (beta 0.29, p = 0.010) (Table 2). Lastly, we analysed the interaction between odour intensity rating and timepoints. The odour intensity rating showed an increasing association with the number of sit-to-stands over time. This was reflected by progressively higher beta values for the interaction term: 0.09 (p = 0.200) at 6 months, 0.13 (p = 0.053) at 12 months, and 0.23 (p = 0.001) at 24 months.

**Table 2:** Results from linear mixed model analyzing the association between olfactory tests (BORG dilution step 6) and physical capacity (number of sit-to-stands). The analysis was done using number of sit-to-stands as the response variable, the participants study ID as a random effect on the intercept and the following fixed effects: timepoints, intensity rating of dilution step 6, age, body mass index, severity of initial infection. Backwards selection was performed to remove non-significant fixed effects with a threshold p-value of 0.05, which resulted in the removal of sex, Charlson comorbidities index and smoking status as covariates.

	Beta	95 % Confidence interval	p-value
**Timepoints**			
**3 months**	–	–	
**6 months**	2.8	0.89, 4.7	**0.004**
**12 months**	4.5	2.6, 6.4	**<0.001**
**24 months**	5.2	3.2, 7.2	**<0.001**

**Age**	−0.5	−0.75, −0.25	**<0.001**

**Intensity rating, dilution step 6**	0.29	0.07, 0.51	**0.01**

**Body mass index**	−1.5	−2.2, −0.77	**<0.001**

**Severity of initial infection**			
**Mild**	–	–	
**Moderate**	−1.4	−12, 9.0	0.8
**Severe**	−15	−28, −2.0	**0.024**

## 4 Discussion

We aimed to investigate the potential long-term association between olfaction and physical capacity in COVID-19 patients. Olfactory impairment measured in an early post-acute phase with the olfactory screening test and rated intensities of n-butanol was strongly associated with low performance on the sit-to-stand test on all time-points. Self-reported olfactory acuity correlated with physical capacity at the 6-month follow-up, while the odour identification test yielded no significant correlations. Further, an impaired sense of smell was occasionally associated with a smaller increase in pulse following the sit-to-stand test (pre to post), but not with ratings of strain or oxygen saturation.

While exploratory, the results were in line with previous research suggesting an association between olfaction and physical capacity (Yuan et al., 2024) and other forms of frailty (Cheng et al., 2024; Yeo et al., 2024). The reason for this relationship still remains elusive. It has been proposed that olfactory impairment may be tied to autonomic dysregulation that stem from a shared underlying pathology involving neural systems important for e.g., baroreflex functioning (Vallée, 2021), which is key to heart rate regulation and postural control. Similarly, deviations in tachykinin (e.g., Substance P) expression, which is related to vagal function, has been described as a putative factor that may link olfactory impairment to fatigue and dysautonomia (Janket et al., 2023/08). Furthermore, case reports suggest that blocking parts of the sympathetic chain with a local anaesthetic ameliorates both persistent anosmia and fatigue in long-COVID (Liu and Duricka, 2022). Our observation that olfactory impairment in the early post-acute phase was associated with lower increase in pulse following the sit-to-stand test, could be interpreted as a blunted sympathetic engagement.

From a wider perspective, post-covid symptoms such as olfactory impairment and reduced physical capacity may be viewed as persistent sickness behaviours in which symptoms normally present during acute infections are manifested over prolonged periods of time (Dantzer et al., 2008/01). Although the mechanisms remain unclear, emerging evidence suggests a connection between post-COVID impairment and broader post-COVID sequelae. Brain imaging studies have revealed both changes in functional networks (Wingrove et al., 2023) and alterations in microstructures associated with olfaction, fatigue, and cognitive functions in patients experiencing post-COVID conditions (Hosp et al., 2024/05). Wingrove et al. (2023) identified functional brain changes in individuals with olfactory impairment, particularly in regions linked to sensory processing and cognition (Wingrove et al., 2023).

An important aspect that cannot be addressed using our current data is whether the olfactory and physical outcomes was due to the COVID infection, or whether some form of prior frailty predisposed the development of the loss of smell. The second scenario implied that the patients with a lower physical capacity did in fact perform close to their baseline, but that this relatively low capacity is a risk factor for olfactory impairment. This aligns with prior evidence suggesting that lower levels of daily physical activity significantly increase the 10-year cumulative risk of developing olfactory impairment (Schubert et al., 2013).

The strength of this study resides in the well-defined cohort with multiple follow-ups over a long time where we have utilized standardised tests. The present study was limited by a small sample size. We furthermore recognise the limitation of using only subjective olfactory assessment at follow-ups and believe that the study would have benefitted from objective olfactory testing at later follow-ups.

## 5 Conclusion

Olfactory impairment 1–3 months after COVID-19 and reduced physical capacity in 1-min sit-to-stand test at 3 months and up to 24 months after infection were significantly correlated in this longitudinal, prospective cohort study. Individuals that in the early post-acute phase after infection had reduced sense of smell were also more likely to have reduced physical capacity after 24 months. The COVID-19 pandemic provided a unique opportunity to explore this connection, highlighting its relevance beyond older populations. These findings deepen our understanding of the relationship between physical performance and olfactory function. Further research with larger cohorts and longitudinal, objective olfactory testing is essential to confirm and expand upon these results.

## CRediT authorship contribution statement

**Christoffer Granvik:** Writing – review & editing, Writing – original draft, Visualization, Project administration, Formal analysis, Data curation. **Alicia Lind:** Writing – review & editing, Supervision, Funding acquisition, Data curation, Conceptualization. **Guilherme W.F. Barros:** Writing – review & editing, Writing – original draft, Methodology, Formal analysis. **Clas Ahlm:** Writing – review & editing, Supervision, Funding acquisition, Conceptualization. **Sara Anderson:** Writing – review & editing, Resources, Investigation. **Linus Andersson:** Writing – review & editing, Writing – original draft, Visualization, Resources, Methodology, Investigation, Conceptualization. **Johan Normark:** Writing – review & editing, Supervision, Resources, Funding acquisition, Conceptualization.

## Data sharing statement

The data that support the findings of this study are available on request from the corresponding author. The data are not publicly available due to privacy or ethical restrictions.

## Funding

The study was funded by grants from 10.13039/100011659Region Västerbotten to AL (RV-992412), JN (RV-993597) and CA (RV-938855), 10.13039/501100003793Swedish Heart-Lung Foundation (20200325 and 20210078, and 20220325 to CA), SciLife Lab COVID-19 research program funded by the 10.13039/501100004063Knut and Alice Wallenberg Foundation (VC-2020-0015 to CA). Swedish Research Council (2016–06514 to J.N.)

## Declaration of competing interest

The authors declare no conflicts of interest related to this study.

## Data Availability

The data that support the findings of this study are available on request from the corresponding author. The data are not publicly available due to privacy or ethical restrictions.
