# Supplementary material for: Olfactory impairment associated with reduced physical capacity 24 months after COVID-19
Source: Brain Behav Immun Health. 2025 Jun 13;47:101032. doi: 10.1016/j.bbih.2025.101032 (PMC12213672; doi:10.1016/j.bbih.2025.101032)
Supplement: Multimedia component 1 [file mmc1.docx]

**Supplementary figure 1.** Study design and physical capacity over time depending on olfactory function after COVID-19. Physical capacity is presented as unadjusted mean values for number of sit-to-stands divided by olfactory acuity.

**
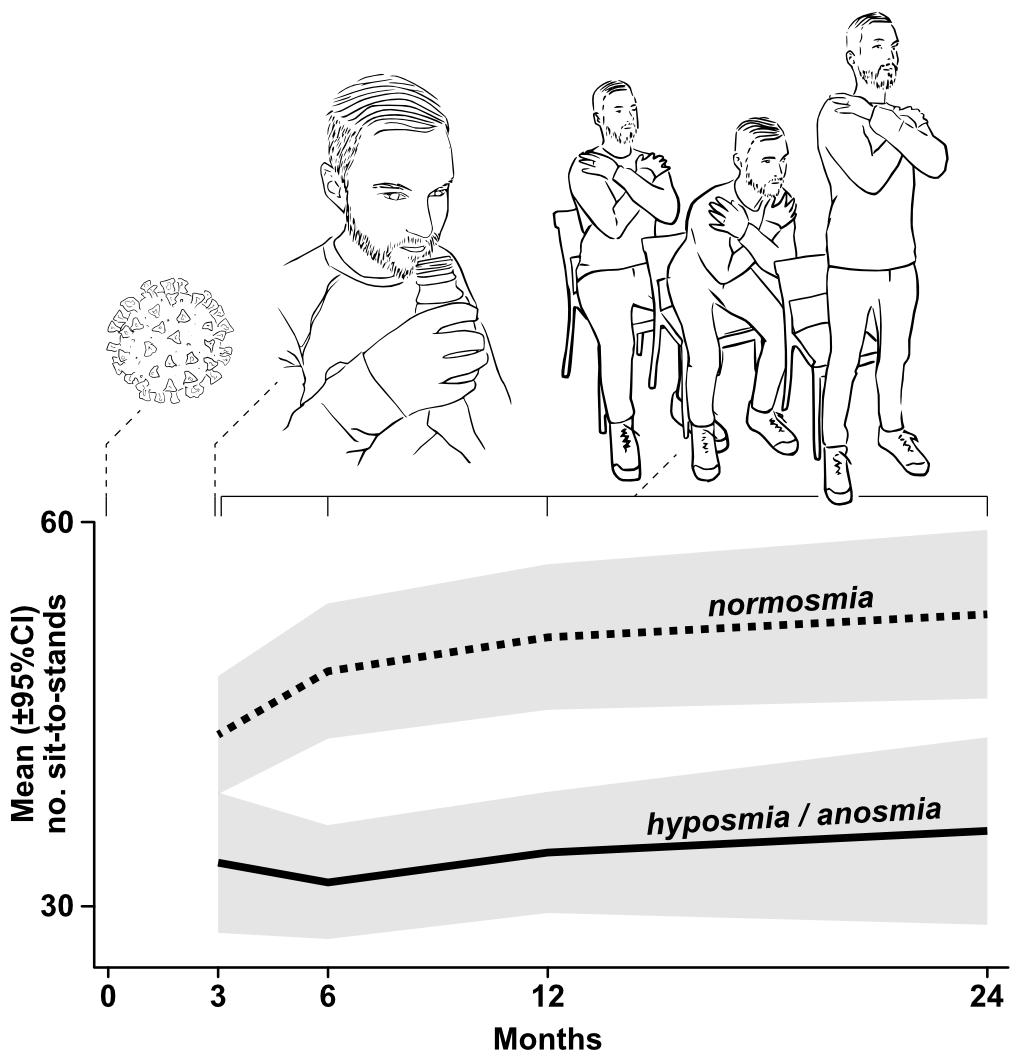
**

**Supplementary table 1.** Demographics and baseline characteristics of the study cohort, divided by sex.

|  | **Men (n = 39)** | **Women (n =24)** | **p-valueª** |
| --- | --- | --- | --- |
| **Age in years – median (IQR)** | 51 (46, 57) | 57 (48, 61) | 0.215 |
| **Body Mass Index – median (IQR)** | 26.2 (23.3, 27.5) | 25.4 (24.5, 26.9) | 0.804 |
| **Comorbidities – n (%)** |  |  |  |
| Cardiovascular disease¹ | 4 (10%) | 1 (4.2%) | 0.641 |
| Chronic lung disease² | 5 (13%) | 5 (21%) | 0.485 |
| Asthma | 4 (10%) | 5 (21%) | 0.283 |
| Brain injury³ | 4 (11%) | 1 (4.2%) | 0.640 |
| Psychiatric illness⁴ | 3 (7.9%) | 3 (13%) | 0.669 |
| Chronic sinuitis | 0 (0%) | 1 (2.5%) | >0.999 |
| **Charlson Comorbidities Index** |  |  | 0.535 |
| 0 – n (%) | 32 (82%) | 18 (75%) |  |
| 1 – 2 mild – n (%) | 7 (18%) | 6 (25%) |  |
| ≥ 3 moderate/severe – n (%) | 0 (0%) | 0 (0%) |  |
| **Smoking status – n (%)⁵** |  |  | 0.504 |
| Non-smoker | 33 (85%) | 17 (77%) |  |
| Former smoker | 6 (15%) | 5 (23%) |  |
| Current smoker | 0 (0.0) | 0 (0.0) |  |
| **Level of education – n (%)⁶** |  |  | >0.999 |
| Lower | 1 (2.6%) | 0 (0%) |  |
| Medium | 12 (31%) | 7 (29%) |  |
| Higher | 26 (67%) | 17 (71%) |  |
| **Hospitalised – n (%)** | 5 (13%) | 4 (17%) | 0.721 |

¹ Ischemic heart disease, congestive heart failure, arrythmias, aortic disease, valvular heart disease or peripheral arterial insufficiency.

² Chronic obstructive pulmonary disease and asthma.

³ Previous brain surgery or head trauma

⁴ History of anxiety, depression or exhaustion syndrome

⁵ Smoking status is missing in 2 patients. The analysis is based on 61 patients.

⁶ Lower: Less than three years beyond Swedish compulsory school. Medium: Three years beyond Swedish compulsory school, but no college or university degree. Higher: University or college degree.

ª Wilcoxon rank sum test; Fisher's exact test; Pearson's Chi-squared test

**Abbreviations:** n, number of patients; IQR, interquartile range.
